# Ga_2_O_3_-Based Optoelectronic Memristor and Memcapacitor Synapse for In-Memory Sensing and Computing Applications

**DOI:** 10.3390/nano14231972

**Published:** 2024-12-08

**Authors:** Hye Jin Lee, Jeong-Hyeon Kim, Seung Hun Lee, Sung-Nam Lee

**Affiliations:** 1 Department of IT & Semiconductor Convergence Engineering, Tech University of Korea, Siheung 15073, Republic of Korea; 2 Department of Nano & Semiconductor Engineering, Tech University of Korea, Siheung 15073, Republic of Korea

**Keywords:** Ga_2_O_3_, memristor, memcapacitor, optoelectronic synapse

## Abstract

This study presents the fabrication and characterization of a dual-functional Pt/Ga_2_O_3_/Pt optoelectronic synaptic device, capable of operating as both a memristor and a memcapacitor. We detail the optimized radio frequency (RF) sputtering parameters, including a base pressure of 8.7 × 10^−7^ Torr, RF power of 100 W, working pressure of 3 mTorr, and the use of high-purity Ga_2_O_3_ and Pt targets. These precisely controlled conditions facilitated the formation of an amorphous Ga_2_O_3_ thin film, as confirmed by XRD and AFM analyses, which demonstrated notable optical and electrical properties, including light absorption properties in the visible spectrum. The device demonstrated distinct resistive and capacitive switching behaviors, with memory characteristics highly dependent on the wavelength of the applied light. Ultraviolet (365 nm) exposure facilitated long-term memory retention, while visible light (660 nm) supported short-term memory behavior. Paired-pulse facilitation (PPF) measurements revealed that capacitance showed slower decay rates than EPSC, suggesting a more stable memory performance due to the dynamics of carrier trapping and detrapping at the insulator interface. Learning simulations further highlighted the efficiency of these devices, with improved memory retention upon repeated exposure to UV light pulses. Visual encoding simulations on a 3 × 3 pixel array also demonstrated effective multi-level memory storage using varying light intensities. These findings suggest that Ga_2_O_3_-based memristor and memcapacitor devices have significant potential for neuromorphic applications, offering tunable memory performance across various wavelengths from ultraviolet to red.

## 1. Introduction

Neuromorphic systems are designed to emulate the human brain, enabling rapid data processing with low power consumption [1,2,3,4]. These systems address the limitations of traditional von Neumann architectures by mimicking the synaptic behavior of biological neural networks. Synaptic devices are crucial in these systems as they adjust their electrical properties in response to various stimuli, allowing for adaptive learning and information processing similar to the human brain. Recent advancements in synaptic devices include developments in optoelectronic, tactile, and chemo-synaptic technologies. In particular, optoelectronic synaptic devices are noteworthy because they emulate biological functions, such as those of the human eye, and offer significant advantages over transitional electrical stimuli due to their low power dissipation [1,2,3]. To effectively mimic visual systems, both sensing and memory functions are essential. This requires the integration of a photodetector and a nonvolatile memory device [5,6,7]. To meet these requirements, optoelectronic memristor and memcapacitor have been proposed [8]. Optoelectronic memristors provide self-adaptive sensing and optical information memory capabilities, but they face limitations, such as excess power consumption and constraints related to the collection area for optoelectronics [9,10,11,12]. In contrast, optoelectronic memcapacitors minimize unnecessary power consumption by utilizing alternative current voltage and exhibit optoelectronic synaptic characteristics through a simple metal–semiconductor–metal (MSM) structure [13,14]. With their high scalability and sensitivity, optoelectronic memcapacitor holds promise for reproducing neuromorphic systems that emulate the functionality of human eyes [15].

The materials used in memcapacitors and memristors include oxide semiconductors, ferroelectrics, and Perovskite [16,17,18,19,20]. Among them, Ga_2_O_3_ has a wide bandgap of 4.9 eV and offers excellent optical, electrical, and thermal stability [21,22]. It has been extensively studied for applications in various electronic and optical devices, including capacitors, photodetectors, and resistive memories [23,24,25]. In particular, Ga_2_O_3_ has been interesting for resistive memory applications due to its ability to switch between high and low resistance states through crystal defects. Moreover, its high photoresponse across visible and ultraviolet (UV) wavelengths, attributed to diverse crystal defect levels, makes Ga_2_O_3_ an excellent candidate for integrating memristor and memcapacitor functionalities [26,27]. This dual capability allows for simultaneous sensing and memory operations, enhancing the overall performance and versatility of neuromorphic systems. However, despite its promising properties, studies specifically addressing Ga_2_O_3_-based memcapacitors remain limited. Furthermore, no studies have explored the simultaneous operation of Ga_2_O_3_ as both a memristor and a memcapacitor, leaving a significant research gap. This study addresses these gaps by fabricating an amorphous Ga_2_O_3_-based MSM structure using radio frequency (RF) sputtering and investigating its resistive and capacitive switching properties under different wavelengths of light. We focused on the optoelectronic synaptic properties arising from these resistive and capacitive behaviors, particularly examining the effects of red, green, blue, and violet light, to evaluate the long-term memory characteristics of photocapacitance.

## 2. Materials and Methods

We grew the Ga_2_O_3_ thin film on a Pt/sapphire template using radio frequency (RF) sputtering. First, the (0001) sapphire substrate underwent a conventional cleaning process. The substrate was sonicated in acetone for 10 min, followed by isopropyl alcohol for another 10 min. Lastly, it was rinsed in deionized water for 10 min to ensure cleanliness. After cleaning, a 50 nm bottom Pt electrode was deposited using a high-purity (99.9%) Pt target. During this deposition, the base pressure inside the sputtering chamber was maintained at 8.7 × 10^−7^ Torr, with an RF power of 100 W, a working pressure of 3 mTorr, and the growth temperature set to room temperature. Next, a 100 nm-thick Ga_2_O_3_ layer was deposited using a high-purity (99.9%) Ga_2_O_3_ target in an Ar atmosphere under similar conditions. Finally, a 50 nm top Pt electrode was deposited using a shadow mask to define the electrode pattern under the same sputtering conditions. This process completed the fabrication of optoelectronic synaptic devices capable of functioning both as a memristor and a memcapacitor. The device consisted of a metal/insulator/metal structure, comprising a bottom Pt electrode, a Ga_2_O_3_ layer serving both resistive and dielectric functions, and a top Pt electrode. This dual functionality was enabled by the unique properties of the Ga_2_O_3_ film, allowing the device to demonstrate both resistive switching and capacitive behavior.

The crystal structure of Ga_2_O_3_ thin film on the bottom Pt electrode was analyzed using an HR-XRD ω/2θ scan (PANalytical, X’Pert Pro MRD, Tokyo, Japan). X-ray photoelectron spectroscopy (XPS) with the ThermoFisher Scientific NEXSA system (Waltham, MA, USA) was employed for chemical bonding analysis, providing composition ratios on the Ga_2_O_3_ surface. The optical band gap of Ga_2_O_3_ was determined through UV–visible optical absorption measurements using a TermoFisher Scientific Evolution 300 spectrophotometer (Waltham, MA, USA). The film structure and surface morphology, including surface grain features, were examined with AFM (NanoFocus my-Scope plus, Seoul, South Korea). Electrical measurements of the memristor were conducted using a Keithley 2614B source meter (Solon, OH, USA), and capacitance was measured using an Agilent 4284A LCR meter (Santa Rosa, CA, USA). To observe the photoreactive properties, light sources of 660, 530, 455, and 365 nm (M660L4, M530L4, M455L4, M365PL1, and THORLABS) were utilized. The optoelectronic synaptic characteristics, such as EPSC_(I)_ and EPSC_(C)_, were evaluated under 0.5 V bias with different light sources. Finally, a comparison and optimization of the optoelectronic synaptic performance based on wavelength were made, focusing on both EPSC_(I)_ and EPSC_(C)_ responses. For the learning experience simulation, the Pt/Ga_2_O_3_/Pt optoelectronic synaptic devices were subjected to 100 cycles of UV light pulses to track the changes in EPSC_(I)_ and EPSC_(C)_. Learning was defined as the EPSC_(I)_ and EPSC_(C)_ reaching 70% of their maximum values while forgetting was measured by the decay of the EPSC_(I)_ and EPSC_(C)_ to 70% of their peak after the stimulation stopped. After this initial phase, a second learning phase was introduced to assess memory retention by reapplying UV light to observe how quickly the device could relearn. For the visual encoding process, a 3 × 3 pixel array was used to simulate visual memory encoding. Different intensities of UV light were applied to each pixel, representing different visual stimuli. The encoded patterns were then stored as variations in EPSC_(I)_ and EPSC_(C)_ and were used to simulate how the device would react to repeated exposure of the same visual stimuli.

## 3. Results and Discussion

**Figure 1:**
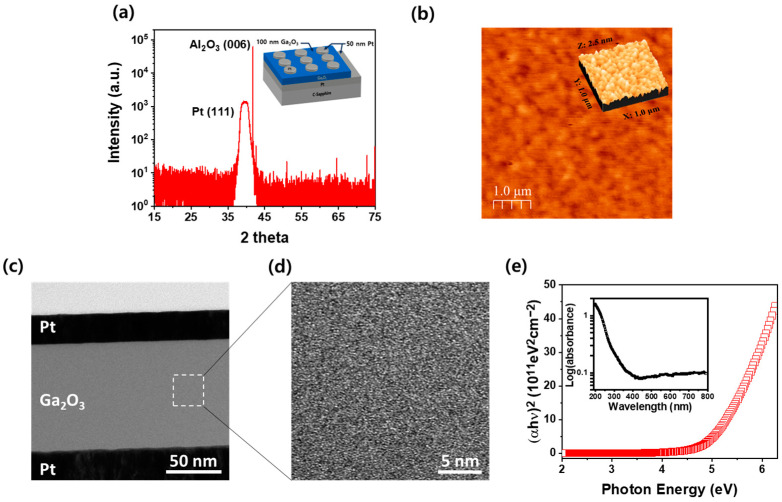
(**a**) XRD ω/2θ scan spectra of the Ga_2_O_3_ film on Pt/sapphire. (**b**) Surface morphologies of the Ga_2_O_3_ film on Pt/c-sapphire measured by AFM. (**c**) Cross-sectional TEM image of the Pt/Ga_2_O_3_/Pt structure. (**d**) Magnified HR-TEM of the Ga_2_O_3_ layer within the white dotted box area of (**c**). (**e**) (αhν)^2^–E plot of the Ga_2_O_3_ thin film, with the inset showing the log(absorbance) wavelength to evaluate the optical absorption of deep levels.

Figure 1a shows a high-resolution X-ray diffraction (HR-XRD) ω/2θ scan spectrum to evaluate the crystalline characterization of Ga_2_O_3_ film grown on a Pt/sapphire template using an RF sputtering system. Two distinct peaks were observed at 41.6° for Al_2_O_3_ (006) and 39.7° for Pt (111), but no peaks corresponding to the Ga_2_O_3_ phase were detected. The inset showed the structure of the Ga_2_O_3_/Pt/sapphire used for XRD measurements. The results confirmed the presence of diffraction peaks corresponding and the sapphire substrate. These results suggest that the Ga_2_O_3_ thin film produced through RF sputtering is amorphous, lacking a specific crystal structure. Figure 1b shows the surface structure of the Ga_2_O_3_/Pt/sapphire obtained from AFM. The surface exhibits extremely small grains, tens of nanometers in size, with a root mean square (RMS) roughness of 0.22 nm. This grain size is significantly smaller than that typically observed in Ga_2_O_3_ thin films grown through crystallization. The grains are so small that it is difficult to discern any crystallization growth, indicating that the Ga_2_O_3_ thin films produced by RF sputtering are amorphous rather than crystalline. This observation is consistent with HR-XRD results as shown in Figure 1a. Figure 1c presents a cross-sectional transmittance electron microscopy (TEM) image of the Pt/Ga_2_O_3_/Pt device as shown in the inset of Figure 1a. The device has a metal–semiconductor–metal (MSM) structure, consisting of a Pt bottom electrode on a c-plane sapphire substrate, a Ga_2_O_3_ layer that simultaneously enables resistive switching and capacitive switching, and a Pt top electrode. Figure 1d shows a high-resolution TEM image of the Ga_2_O_3_ region in the structure, revealing a lack of regular atomic arrangement typical of crystalline thin films. Instead, a random, amorphous structure is observed, indicating that the sputter-grown Pt/Ga_2_O_3_/Pt thin films used in this study are entirely amorphous. Figure 1e shows the (αhν)^2^ of the Ga_2_O_3_ thin film as a function of photon energy for a sputter-grown Ga_2_O_3_ thin film. The absorption coefficient was calculated using the equation: αhν = B(hν − E_g_)^1/2^, where α is the absorption coefficient, h is the Plank’s constant (4.135 × 10^−15^ eVs), ν is the frequency (s^−1^), B is a constant, and E_g_ is the energy band gap (eV) [28]. The optical bandgap of the Ga_2_O_3_ film, calculated to be 4.87 eV, was determined by extrapolating the linear portion of the absorption curve to the energy axis. This value is lower than the 4.9 eV bandgap of crystalline β-Ga_2_O_3_. Despite the amorphous nature of the Ga_2_O_3_ film, the presence of a distinct optical bandgap demonstrates its ability to absorb photons effectively. In addition, high absorption in the UV region suggests that the Ga_2_O_3_ thin film could be well-suited for use in UV-sensitive memristors and memcapacitors for optoelectronic synaptic devices. To identify defect-related deep levels within the bandgap, a plot of log(absorbance) versus photon wavelength is shown in the inset of Figure 1e. The plot suggests the presence of deep levels in the Ga_2_O_3_ film that enable light absorption in the visible range, between 2.0 eV and 3.0 eV. The relatively high absorption in the UV range (3.0 to 3.5 eV) and the weaker absorption in the visible range (2.0 to 3.0 eV) indicate that these features are present in sputter-grown Ga_2_O_3_ films. Typically, absorption levels around 2.5 eV and 3.0 eV are associated with gallium vacancy (V_Ga_)-related defects, while absorption around 3.5 eV is attributed to oxygen vacancy (V_O_)-related defects [29]. The bandgap of amorphous Ga_2_O_3_ thin films is reduced due to various crystal defects, allowing them to absorb light through shallow and deep levels [30]. This enables the films to function as photosensitive devices capable of absorbing not only high-energy ultraviolet light but also lower-energy visible light.

**Figure 2:**
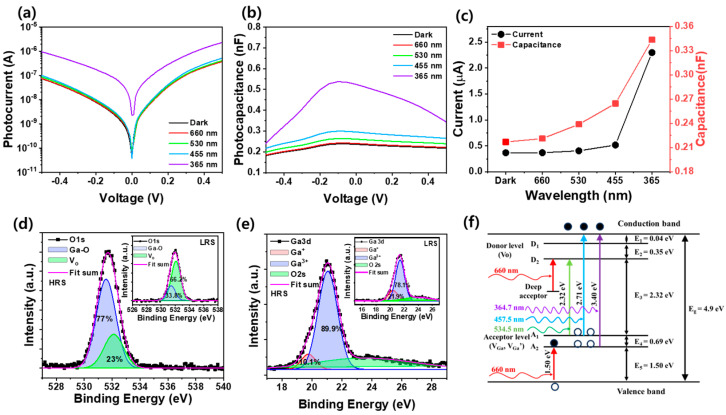
(**a**) Photocurrent and (**b**) photocapacitance of Pt/Ga_2_O_3_/Pt memcapacitor with different applied wavelength. (**c**) Trend in current and capacitance according to applied wavelength. XPS spectra of (**d**) O 1s and (**e**) Ga 3d in a Pt/Ga_2_O_3_/Pt memristor in a HRS. (**f**) Schematic of photoreaction mechanism of Ga_2_O_3_ bandgap [33,34]. Insets of (**d**,**e**) show O 1s and Ga 3d spectra in a Pt/Ga_2_O_3_/Pt memristor in LRS, respectively.

Figure 2a,b show the photocurrent and photocapacitance of a Pt/Ga_2_O_3_/Pt optoelectronic synaptic device as a function of the applied voltage in the dark state and under illumination with light at wavelengths of 365, 450, 530, and 660 nm, respectively. Both graphs demonstrate a slight increase in photocurrent and photocapacitance. As the visible light wavelength decreases, a significant increase is observed when 365 nm UV light is applied. Figure 2c illustrates the variation of the photocurrent and photocapacitance with increasing wavelength of light from 365 nm to 660 nm applied at 0.5 V. The dark current is 367 nA, which increases from 370 nA to 516 nA as the excitation wavelength decreases from 660 nm to 450 nm. A substantial rise in photocurrent to 2.29 μA is observed when 365 nm UV light is applied. It indicates that at lower energy levels, such as the 660 nm red light, fewer photoexcited carriers are formed, while at higher energy levels, such as the 365 nm UV light close to the bandgap, more photoexcited carriers can be generated. This is likely due to the high absorption in the UV region, as shown in Figure 1e. Moreover, the dark capacitance at 0.5 V is 0.21 nF but increases from 0.22 nF to 0.26 nF as the excitation wavelength decreases from 660 nm to 450 m, and significantly increases to 0.34 nF with the application of 365 nm UV light. The photocapacitance of Pt/Ga_2_O_3_/Pt devices can be attributed to the generation and trapping of photoexcited carriers under illumination. This effect is much stronger under UV light due to the high energy that efficiently generates carriers across the bandgap of Ga_2_O_3_, whereas visible light causes smaller changes due to lower energy photon interactions, mainly associated with defects or sub-bandgap states. Therefore, it is believed that Ga_2_O_3_ can be effectively photoexcited by UV wavelength with high photon energy. Figure 2d,e show the O 1s and Ga 3d spectra of Pt/Ga_2_O_3_/Pt optoelectronic synaptic device with HRS, respectively. The chemical composition of the Ga_2_O_3_ thin film was analyzed using XPS, focusing on the O 1s and Ga 3d peaks. The O 1s peak is divided into two components: the Ga–O peak at 531.7 eV, which corresponds to the binding energy of oxygen in the Ga_2_O_3_ lattice, and the V_o_ peak at 532.1 eV, which is associated with oxygen vacancies. The proportion of oxygen vacancies on the surface was determined by calculating the ratio of the V_o_ peak to the total area of the O 1s peak, which was found to be 23%. This indicates a significant presence of oxygen vacancy point defects in the amorphous Ga_2_O_3_ thin film. Such defects contribute to the formation of the amorphous structure, as opposed to the crystalline form of Ga_2_O_3_, and lead to enhanced conductivity and alterations in the bandgap. The inset of Figure 2d shows the O 1s spectrum of the Pt/Ga_2_O_3_/Pt optoelectronic synaptic device in LRS. Analysis of the O 1s XPS spectra in the LRS revealed that the Ga-O peak accounted for 33.8%, while oxygen vacancies (V_o_) contributed 66.2%. During the transition from HRS to LRS, the ratio of oxygen vacancies increased significantly from 23% to 66.2%, indicating that oxygen vacancies play a critical role in forming the conductive filament channel responsible for resistive switching. In addition, as shown in Figure 2e, the Ga 3d peak consists of contributions from O 2s, Ga 3d (Ga^3+^), and Ga 3d (Ga^1+^). The Ga 3d peak can be decomposed into Ga^3+^ and Ga^1+^ peaks, corresponding to binding energies of 21.0 eV and 19.7 eV, respectively [31]. The Ga^3+^ state represents gallium in its fully oxidized form, which is typical in stoichiometric Ga_2_O_3_ and forms stable bonds with oxygen atoms in the crystal lattice. The Ga^+^ state corresponds to gallium in a reduced oxidation state, such as the Ga_2_O phases, indicating the presence of gallium with a lower oxidation state than Ga^3+^. The Ga^3+^ peak has an area 8.7 times larger than the Ga^+^ peak, indicating that the amorphous gallium oxide is predominantly composed of Ga_2_O_3_. The presence of the Ga^+^ peak suggests that oxygen vacancies or gallium interstitials are present, which can reduce the effective oxidation state of gallium in specific regions [32]. Therefore, it is evident that gallium exists primarily in the fully oxidized Ga^3+^ state, but also in a reduced Ga^+^ state, likely due to the presence of oxygen vacancies or interstitial defects in the lattice. Moreover, the inset of Figure 2e illustrates the Ga 3d peaks of Pt/Ga_2_O_3_/Pt optoelectronic synaptic device in the LRS. As the device transitions from HRS to LRS, the Ga^+^ ratio increases from 19.1% to 21.9%, while the Ga^3+^ ratio decreases from 89.9% to 78.1%. This indicates that Ga 3d analysis reflects an increase in oxygen vacancies during the transformation process from HRS and LRS, which is consistent with the O 1s results shown in Figure 2d. Figure 2f shows the shallow and deep levels corresponding to various defects within the Ga_2_O_3_ band gap, as well as the photocarrier excitation process as a function of the applied wavelength. It is well known that the donor levels, generated by oxygen vacancies in Ga_2_O_3_ thin films, are located at approximately 0.04 eV and 0.39 eV below the conduction band. In addition, acceptor levels formed by gallium vacancies and gallium-oxygen vacancies are positioned around 1.5 eV and 2.19 eV above the valence band [33,34]. Compared to the optical band gap of 4.87 eV for the Ga_2_O_3_ thin film, as shown in Figure 1d, the energy corresponding to wavelengths from 365 nm to 660 nm (3.4 eV to 1.9 eV) is lower, which is insufficient to directly photoexcite Ga_2_O_3_ across its band gap. However, due to the presence of numerous shallow levels, carrier excitation is still possible with energies lower than the band gap of the Ga_2_O_3_ thin film. As a result, additional charge carriers can be generated not only at UV wavelengths but also at visible wavelengths, leading to an increase in both current and capacitance. When light is applied, holes at the valence band and acceptor level are excited at the donor levels and conduction band. This process generates electron–hole pairs, causing photocurrent to flow as the carriers move toward the Pt electrode. Therefore, the observed increase in current upon light exposure can be attributed to the increase in free carriers generated by these defective states. The increase in capacitance observed when light is applied can be attributed to the release of the trapped carriers. When light illuminates the Ga_2_O_3_ thin film, electrons trapped in defects such as oxygen vacancies are liberated. This process forms empty trap sites, which contribute to a reduction in the Schottky barrier height and an additional potential. The liberated carriers enhance the total amount of accumulated charge, leading to an increase in capacitance [35]. It is believed that longer wavelengths of excitation light generate fewer photoexcited carriers due to their lower excitation energy. This is because higher photon energy, associated with shorter wavelengths, is more effective at promoting electrons from the valence band to the conduction band, thereby increasing the number of photoexcited carriers. As a result, the photocurrent and photocapacitance values of the Pt/Ga_2_O_3_/Pt optoelectronic synaptic device increase with the excitation light in the following order: red (660 nm), green (530 nm), blue (450 nm), and UV (365 nm), as shown in Figure 2c.

**Figure 3:**
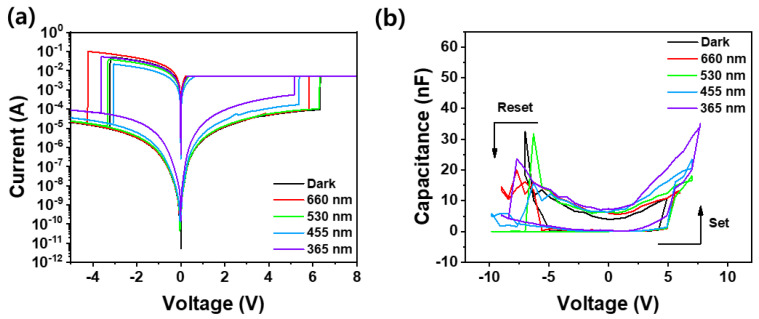
(**a**) I–V resistive switching and (**b**) C–V capacitive switching behaviors of Pt/Ga_2_O_3_/Pt optoelectronic synaptic device by applying the different exposure lights ranging from 365 nm to 660 nm.

Figure 3a,b illustrate the bipolar resistive and capacitive switching behaviors of the I−V and C−V characteristics in the Pt/Ga_2_O_3_/Pt optoelectronic synaptic device, both in darkness and under varying light wavelengths from 365 nm to 660 nm. The resistive switching mechanism operates in four voltage stages. First, as the voltage increases from 0 V to 8 V, conductive filament channels form in the Ga_2_O_3_ film, transitioning the device from a high-resistance state (HRS) to a low-resistance state (LRS) in the “set” phase. These filaments enable current conduction. When the voltage is reduced back to 0 V, the filaments remain intact, keeping the device in the LRS and preserving the low-resistance memory. In the next stage, as the voltage reverses from 0 V to −5 V, the conductive filaments break down, switching the device back to the HRS in the “reset” phase. As the voltage sweeps back from −5 V to 0 V, the device stays in the HRS without reforming the filaments, maintaining its HRS. During the HRS phases, the photocurrent shows a clear dependence on the wavelength of light, increasing as the wavelength shortens. This behavior, shown in Figure 2a, emphasizes the optoelectronic characteristics of the synaptic device. For visible light wavelengths, ranging from red to blue, shorter wavelengths provide higher photon energy, which leads to an increase in photoexcited carriers. However, this increase is relatively modest, and the photocurrent remains significantly higher under UV light at 365 nm compared to visible light. This is because, as shown in Figure 2f, visible light does not generate substantial excitation due to its limited penetration depth and its inability to effectively excite the deep-level states associated with oxygen vacancy defects in Ga_2_O_3_ film. In contrast, UV light penetrates deeper into the material, allowing it to excite these deep-level states, resulting in a much larger photocurrent. In the LRS, however, the photocurrent shows little sensitivity to the wavelength of the excitation light. This is because, in the LRS, the current is primarily conducted through the robust filament channels formed with the Ga_2_O_3_ thin film. The contribution of photoexcited carriers becomes negligible in comparison to the dominant carrier transport through these conductive filaments, which effectively mask any impact that the excitation light might have on the overall current. Figure 3b shows the capacitive switching properties of the Pt/Ga_2_O_3_/Pt device through C-V sweeps, measured under various light wavelengths (365 nm to 660 nm) and in darkness. Under 365 nm UV light, the device exhibits the highest capacitance, especially during the set process (positive voltage region). The gradual increase in capacitance at lower voltages suggests that UV light generates more photoexcited carriers, increasing capacitance in the LRS. This is consistent with the results that UV light excites deeper levels, enhancing carrier density as shown in Figure 2a. As the wavelength increases from 450 nm to 530 nm, capacitance decreases but still shows significant capacitive switching, indicating that shorter visible wavelengths can influence carrier generation, though less effectively than UV light. At 660 nm, capacitance is much lower, with a reduced response in both the set and reset regions, implying that longer wavelengths (closer to red) produce fewer photoexcited carriers, and thus less capacitive enhancement. The C-V measurement is less stable compared to resistive switching (RS) behavior in Figure 3a, likely due to the inability to control high current compliance in the C-V measurement. Similar to the RS characteristics, the transition from HRS to LRS corresponds to increased capacitance. Oxygen vacancies in the Ga_2_O_3_ film are likely concentrated during conductive filament formation, increasing dipole carriers and capacitance. UV light further enhances this effect by generating more photoexcited carriers, which migrate around oxygen vacancies, amplifying capacitance in the LRS. Thus, as the excitation wavelength increases, capacitance in the LRS decreases.

**Figure 4:**
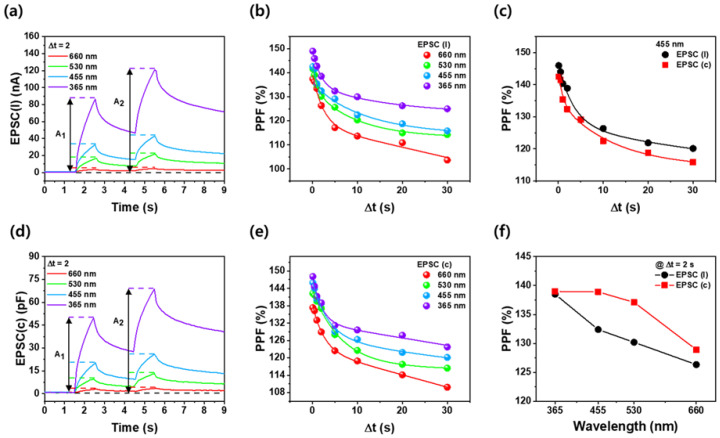
(**a**) EPSCs_(I)_ and (**d**) EPSC_(C)_ of Ga_2_O_3_ optoelectronic synaptic devices measured at 0.5 V under different applied wavelengths using two consecutive light pulse injections of 1.0 s exposure, followed by 2.0 s off. PPF values for (**b**) EPSC_(I)_ and (**e**) EPSC_(C)_ at various time intervals and applied wavelengths. (**c**) Comparison of PPF between EPSC_(I)_ and EPSC_(C)_ at 455 nm over time interval. (**f**) Comparison of PPF between EPSC_(I)_ and EPSC_(C)_ as a function of applied wavelength at a time interval of 2.0 s.

Figure 4a,d show the photosynaptic time at two pulses of 1.0 s photoexcitation followed by 2.0 s darkness, plotted as excitatory postsynaptic current (EPSC_(I)_) and excitatory postsynaptic capacitance (EPSC_(C)_) as a function of wavelength. Paired-pulse facilitation (PPF), a measure of short-term plasticity in optoelectronic synaptic devices, quantifies the connection strength of a synapse by calculating the ratio of the second pulse (A_2_) to the first pulse (A_1_). This method confirmed short-term plasticity via red light excitation in the UV [36,37]. Both EPSC_(I)_ and EPSC_(C)_ exhibited higher optical excitation and slower decay at shorter wavelengths. Figure 4b,e display the PPFs for varying time intervals (Δt) from 0.1 s to 30 s as a function of wavelength in EPSC_(I)_ and EPSC_(C)_, respectively. PPF decreased with increasing Δt across the entire wavelength range, aligning with the time dependence of PPF in photosynaptic devices and biological synapses. For both EPSC_(I)_ and EPSC_(C)_, PPF values increased with shorter excitation wavelengths. Specifically, as the wavelength decreased from 660 nm to 365 nm, PPF increased from 111% to 126% for EPSC_(I)_ and from 114% to 128% for EPSC_(C)_ at Δt = 20 s. This indicates enhanced short-term plasticity at shorter wavelengths, suggesting improvements in both short- and long-term memory characteristics. Figure 4c compares the PPF values of EPSC_(I)_ and EPSC_(C)_ at a wavelength of 455 nm, confirming that EPSC_(C)_ exhibited a higher PPF value than EPSC_(I)_ across all Δt. In addition, Figure 4f demonstrates that EPSC_(C)_ exhibits a higher PPF value than EPSC_(I)_ across all wavelength bands at Δt = 2.0 s. This indicates that EPSC_(C)_ has better short-term plasticity compared to EPSC_(I)_ across all wavelengths. The difference in PPF values is attributed to the decay mechanisms following LED stimulation in the Ga_2_O_3_ thin film. When light is applied to Pt/Ga_2_O_3_/Pt structure, photons excite electrons in the conduction and acceptor levels, forming electron–hole pairs and generating a photocurrent. Once the external stimulus is removed, the generated free carriers recombine, leading to a reduction of photocurrent. For capacitance, the generation of electron–hole pairs increases the movable effective charge density within the Ga_2_O_3_ film. Additionally, electrons trapped at the interface are released by the external stimulus, which increases the total charge. The relationship, C = Q/V (Q is charge and V is voltage), indicates that capacitance increases with accumulated charge. After the external stimulus is removed, the released carriers are re-trapped by defects, leading to a decrease in capacitance due to the reduction in additional carriers. The higher PPF value of EPSC_(C)_ compared to EPSC_(I)_ is due to the distinct behaviors of charge carriers. Rapid recombination in EPSC_(I)_ leads to a swift decline, as it is sensitive to immediate carrier availability. In contrast, capacitance can store charge more effectively, with some carriers becoming trapped in defect states [38]. This prevents immediate recombination and results in a slower decay of capacitance, allowing it to maintain higher levels over time. Thus, capacitance demonstrates a more robust long-term memory effect, enhancing its overall characteristics.

**Figure 5:**
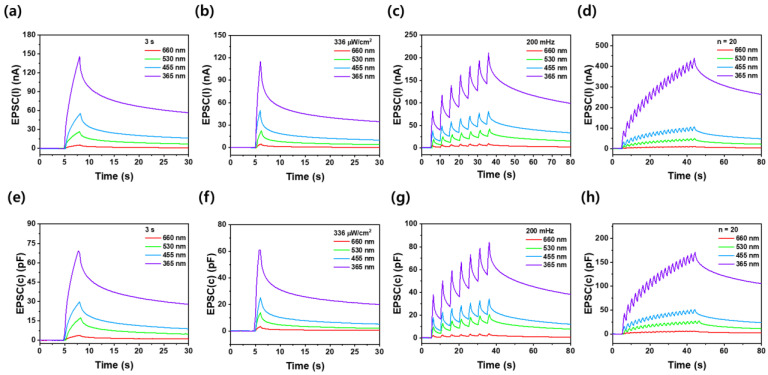
Synaptic characteristics of (**a**–**d**) EPSCs_(I)_ and (**e**–**h**) EPSCs_(C)_ under varying conditions: (**a**,**e**) 3.0 s exposure duration, (**b**,**f**) light power intensity of 336 µW/cm^2^, (**c**,**g**) light input frequency of 200 mHz, and (**d**,**h**) 20 exposure cycles at different wavelengths.

In Figure 5a–h, the effects of wavelength energy on EPSC_(I)_ and EPSC_(C)_ are presented. The photosimulation conditions were evaluated based on duration, light intensity, frequency, and the number of exposures. In the Ga_2_O_3_ optoelectronic synapse device, a duration of 3.0 s, light intensity of 336 µW/cm^2^, frequency of 200 mHz, and exposure cycles of 20 indicate that optical potentiation effectively responds to decreasing wavelengths. During the application of light of 3.0 s, the wavelength decreased from 660 nm to 365 nm, resulting in an increase in EPSC_(I)_ from 5.4 nA to 146 nA and an increase in EPSC_(C)_ from 3.9 pF to 69.3 pF. At 30 s after stopping LED exposure, EPSC_(I)_ changed from 0.9 nA to 56.8 nA, while EPSC_(C)_ changed from 1.1 nF to 28 nF. These results confirm that higher energy wavelengths retain greater EPSC_(I)_ and EPSC_(C)_ characteristics even after optical excitation ceases. Therefore, integrating these properties into neuromorphic computing systems is advantageous with shorter wavelengths providing better long-term memory (LTM) characteristics through optical stimulation and output [39,40]. Additionally, a single Pt/Ga_2_O_3_/Pt device demonstrates four distinct photoexcited long-term memory states corresponding to the applied wavelength. By diversifying the photon conditions for each wavelength band, it becomes possible to adjust the levels expressed in synapses exponentially, expanding the diversity of information storage and enhancing the efficiency and performance of synaptic devices.

**Figure 6:**
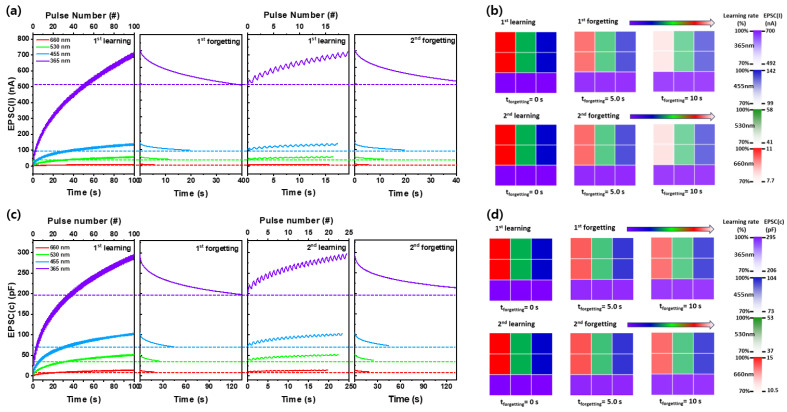
Learning and forgetting experience behaviors under pulsed light stimulation (0.5 s pulse width and 50% duty cycle) measured by (**a**) EPSC_(I)_ and (**c**) EPSC_(C)_. Mimicking human visual memory processes through the learning and forgetting experience based on (**b**) EPSC_(I)_ and (**d**) EPSC_(C)_.

Figure 6a,c present the learning-experience simulation results of EPSC_(I)_ and EPSC_(C)_ for wavelength dependence photoexcitation in a Pt/Ga_2_O_3_/Pt optoelectronic synaptic device. Optical potentiation was achieved by applying 100 pulse cycles (0.5 s pulse width, 50% duty cycle) across four wavelength bands, from UV to red, to obtain the maximum EPSC_(I)_ and EPSC_(C)_. A threshold of 70% of the maximum EPSC_(I)_ and EPSC_(C)_ was set for learning and forgetting. The number of pulses required for the first learning cycle of EPSC_(I)_ across all wavelengths was approximately 60 pulses, while the second learning cycle required significantly fewer pulses, around 18 pulses. Although the pulse count varied slightly with wavelength, it remained consistent as the increase from 70% to the maximum value followed a similar pattern. The second learning cycle required fewer pulses, indicating that the number of pulses needed to reach the desired EPSC_(I)_ and EPSC_(C)_ decreased with repeated training. As the wavelength decreased from 660 nm to 365 nm, the time for 70% forgetting increased from 5.0 s to 39 s for EPSC_(I)_ and from 18 s to 133 s for EPSC_(C)_. This suggests that learning is less effective, and retention is shorter at longer wavelengths (red light) compared to shorter wavelengths (UV light). Notably, at 660 nm, EPSC_(I)_ declined to 70% within just 5.0 s after the first optical learning, indicating rapid forgetting at longer wavelengths. In contrast, with 365 nm UV stimuli, it took 39 s for the EPSC_(I)_ to decline after the first learning process, but after the second learning process, the EPSC_(I)_ remained at 72% of its peak after the same 39 s, about 2.0% higher than after the first learning. This indicates that memory retention improved with the second learning compared to primary learning. Simulation of learning using EPSC_(C)_ also showed that both the maximum EPSC_(C)_ and the number of pulses required to reach it during the first learning cycle decreased as the wavelength increased. This indicates that as the wavelength increases, light energy decreases, resulting in a slower rate of EPSC_(C)_ increase and faster learning at longer wavelengths. In the forgetting process following photosimulation, UV light led to a 70% reduction in EPSC_(C)_ after 133 s, while 660 nm red light exhibited forgetting after just 18 s. This shows that UV light, with its higher energy, leads to slower forgetting compared to red light. In the second forgetting process, red light led to an EPSC_(C)_ retention of 70.5% after 18 s, while 365 nm UV light resulted in a higher retention of 84.7% after 133 s. This suggests that repeated learning improves retention across all wavelengths, from UV to red. However, while learning occurs faster under low-energy, long-wavelength light, forgetting characteristics are better at shorter, high-energy wavelengths. This slower forgetting rate is attributed to the re-trapping of photoexcited carriers at defect levels within the Ga_2_O_3_ material, which delays their recombination. This suggests that EPSC_(C)_, compared to EPSC_(I)_, is more suitable for LTM applications, as it allows memory to last longer without easily fading.

Figure 6b,d present a simulated visual layout of a 3 × 3 pixel array, representing two iterations of learning using EPSC_(I)_ and EPSC_(C)_ values under UV, blue, green, and red light stimulation. Two devices from a selection of nine on a wafer were tested, with the EPSC_(I)_ and EPSC_(C)_ values from pulse inputs encoding the pixel color contrast, as shown in the scale bar on the right. The intensity of the pixel colors reflects the values defined in Figure 6a,c. Figure 6b show that after the first training with varying wavelengths, memory decays faster with red light, where the photostimulus memory fades after 6.0 s. Longer wavelengths exhibited slower memory decay, as indicated by darker colors, suggesting better learning retention. In contrast, the second learning process, EPSC_(I)_ and EPSC_(C)_ decayed more slowly, resulting in darker images after the same period, as shown in Figure 6b,d. This trend was observed for both EPSC_(C)_ and EPSC_(I)_, indicating that higher-energy 365 nm UV light produces better learning memory properties, potentially enhancing the LTM characteristics of optoelectronic synaptic devices. Furthermore, EPSC_(C)_ demonstrated superior memory retention, maintaining learning properties for up to 18 s, more than double the forgetting time of EPSC_(I)_, which was 8.0 s.

## 4. Conclusions

We successfully fabricated a Pt/Ga_2_O_3_/Pt optoelectronic synaptic device using RF sputtering, confirmed by XRD and AFM analyses, which revealed the amorphous nature and light absorption properties of the Ga_2_O_3_ film. The Ga_2_O_3_ thin film features shallow and deep defect levels, including donor levels from oxygen vacancies and acceptor levels from gallium vacancies. While the band gap of Ga_2_O_3_ film is 4.87 eV, the presence of shallow levels allows carrier excitation even with light wavelengths below the band gap. As a result, current and capacitance increase with light exposure, with photocurrent and photocapacitance values increasing in the order: red, green, blue, and UV light. The device demonstrated promising optoelectronic synaptic characteristics, exhibiting distinct memory behaviors based on the applied light wavelength. Shorter wavelengths, particularly at 365 nm, were effective in supporting long-term memory retention, while longer wavelengths (660 nm) facilitated short-term memory functions. Paired-pulse facilitation (PPF) experiments revealed that capacitance-based measurements (EPSC_(C)_) offered superior memory stability compared to current-based EPSC_(I)_, primarily due to the slower decay of capacitance resulting from carrier trapping and detrapping at the insulator interface. This finding highlights the potential of capacitance as a reliable parameter for sustaining memory retention. Furthermore, visual memory simulations demonstrated the capacity of the device to encode and retain visual stimuli using a 3 × 3 pixel array, confirming the potential of the device for image processing applications. Overall, this study demonstrates that dual-functional Ga_2_O_3_-based optoelectronic synaptic devices, functioning as both memristors and memcapacitors, can serve as multi-level storage elements. These devices respond to light wavelengths ranging from ultraviolet to visible, allowing for versatile memory retention and dynamic storage capabilities. By adjusting the applied wavelength, the devices exhibit tunable memory properties, making them promising candidates for advanced neuromorphic computing and multi-state memory systems.

## Data Availability

The data presented in this study are available on request from the corresponding author. The data are not publicly available due to privacy concerns.
